# Evaluation of candidate RT-qPCR reference genes in the aging African turquoise killifish brain

**DOI:** 10.1016/j.nbas.2025.100151

**Published:** 2025-09-09

**Authors:** Emily Whisenant, Arne C. Lekven

**Affiliations:** Department of Biology and Biochemistry, University of Houston, Houston, TX, USA

**Keywords:** RT-qPCR, Reference gene, Gene expression, Sexual dimorphism, *Nothobranchius furzeri*, Aging, Brain aging, Neurodegeneration

## Abstract

Reference genes (RGs) are typically used to normalize gene expression from RT-qPCR experiments. However, the expression of commonly used RGs can vary across different physiological conditions, such as aging, and potentially lead to inaccurate interpretations of results. In African turquoise killifish (*Nothobranchius furzeri*), the stability of reference genes has not been evaluated during aging. Here, we evaluate six candidate reference genes used in other models of aging (*actb*, *cyc1*, *gapdh*, *gusb*, *oaz1a*, and *tbp*) and examine their brain expression stability in adult males and females from young (10 weeks post-hatching) to old (25 weeks post-hatching). To examine RG stability, we used a combination of summary statistics based on analyses of Cq values, normalized fold change of tyrosine hydroxylase (*th*), and available computational programs. Overall, we found that *cyc1*, *oaz1a*, and *gusb* were the most stable reference genes during aging across both sexes, with specific rankings reflecting sex-dependent differences, while *gapdh* and *actb* were the least reliable. Importantly, when *th* expression was normalized to our selected RGs, we found that only female samples had an age-related decrease in expression, and expression analysis was highly dependent on the choice of reference gene. Taken together, our findings provide the first systematic evaluation of RG stability in the killifish brain and highlight *cyc1*, *oaz1a*, and *gusb* as reliable RGs for studies of aging. We recommend that future studies use at least two of these RGs in combination for accurate normalization and evaluate RGs for selected experimental conditions within the framework established in this study.

## Introduction

Aging is accompanied by widespread changes in gene expression that can affect brain function, plasticity, and vulnerability to disease [1]. Changes in gene expression profiles in aging occur in various tissue types [2] and in different model systems [[3], [4], [5], [6]]. However, these patterns are not universally conserved, and there are species-specific aspects of aging [7]. Though many transcripts change throughout aging, one commonly studied gene affected by aging is tyrosine hydroxylase (*th*). The *th* gene codes for the rate-limiting enzyme in catecholamine synthesis [8]. In Parkinson’s disease, an age-associated neurodegenerative disorder, *TH* expression decreases throughout aging. This leads to a loss of TH-associated neurons, corresponding accumulation of α-synuclein fibrils, and formation of Lewy bodies [9,10]. Although this mechanism is typically associated with age in humans, young animal models of Parkinson’s disease can replicate this mechanism through genetic mutations to Parkinson’s associated genes, neurotoxin exposure, or overexpression of α-synuclein [11]. In African turquoise killifish (*Nothobranchius furzeri),* there is a progressive loss of Th^+^ neurons that occurs spontaneously as the fish ages, without genetic or environmental manipulation [12], though this phenotype is reportedly strain-specific [13]. Additional studies investigating *N. furzeri* aging found further age-related changes in gene expression in the brain, such as cell-specific decreases in proliferative markers [[14], [15], [16]] reminiscent of changes seen in other model organisms [17].

To quantify age-associated gene expression changes, a frequently used technique is RT-qPCR. This technique measures transcripts of interest by normalizing expression to reference genes (RGs) or “housekeeping genes”. For reliable normalization, the ideal RG is expressed steadily across all experimental conditions. For example, in studies investigating changes in *th* across the *N. furzeri* aging brain, the optimal RG would have unchanging levels of expression at all considered ages. Notably, there is no universally perfect reference gene, and a variety of factors, such as biological sex, should be considered when analyzing candidate RGs, particularly regarding aging. In aging mice, there is a sex-dependent difference in aging muscle tissue; in this case, some RGs are stable in one sex across aging, but not in another [18], highlighting the need to select the appropriate RG for individual studies. Sex is also a known factor in brain aging and the development of neurodegenerative disorders [19]. Thus, both sex and age must be considered as factors when evaluating RG stability for transcript normalization in the adult brain. Despite its growing use as a model of aging, there is currently no study measuring the efficacy of RGs in RT-qPCR in *N. furzeri*.

To address this gap, we investigated the expression characteristics of reference genes commonly used in brain aging studies in other model systems. In our study, we tested the stability of six candidate reference genes, β-actin (*actb*), cytochrome C1 (*cyc1*), glyceraldehyde-3-phosphate dehydrogenase (*gapdh*), β-glucuronidase (*gusb*), ornithine decarboxylase antizyme 1a (*oaz1a*), and TATA-box binding protein (*tbp*)*,* in the brains of male and female *N. furzeri* at two different ages. Some commonly used RGs, such as *ActB* or *Gapdh*, have reported variable activity during brain aging and neurodegenerative disorders, leading to potential compromised reliability of normalized target gene expression [20]. Our other putative RGs (*Cyc1, Gusb, Oaz1a,* and *Tbp*) are found to be reliable in analyses of other aging models [[20], [21], [22]]. We hypothesized that *cyc1, gusb, oaz1a,* and *tbp* would be more stable than *actB* and *gapdh*, aligning with patterns seen in other model systems. Our results support this, and suggest that *cyc1*, *oaz1a*, and *gusb* are stable reference genes in both sexes throughout progressive *N. furzeri* neurodegeneration.

## Methods

### Fish care

GRZ strain *N.furzeri* were raised on a 12/12 light cycle and maintained as described in Nath et al. [23]. Adult fish were euthanized at 10 or 25 weeks post-hatching in 0.1 % tricaine solution for 10 min. These time points were selected as important ages for neuronal degeneration and in the fish growth rate [[12], [13], [14], [15], [16], [17], [18], [19], [20], [21], [22], [23], [24]]. Fish were decapitated and brains were immediately dissected and homogenized in 250 μL of TriZol (Invitrogen). Lysate was stored at −80 °C until further processing.

### RNA purification, cDNA synthesis, and RT-qPCR

RNA was purified from TriZol lysate as per the manufacturer’s protocol. To synthesize first-strand cDNA, we used 250 ng of purified RNA and the SuperScript IV First Strand Synthesis System (Invitrogen). The resulting product was diluted 1:10 and 1 µL was used in a 12 µL reaction with Power SYBR Green PCR Master Mix (Thermo Fisher) and cycled with a Q qPCR machine (Quantabio). Cycling parameters followed manufacturers’ suggestions and are listed as follows: 10 min activation at 95 °C (1x), 15 s at 95 °C and 60 s at 60 °C (40x). Melt curves were generated post-amplification by melting from 72 °C to 95 °C at a rate of 0.3 °C/sec. Each sample consisted of one whole adult brain. For each sample, we ran three technical replicates to generate an average Cq value for subsequent evaluation.

### Primer design

Primers for *cyc1* (XM_015958440.3), *gusb* (XM_015961804.3), *gapdh* (XM_015950201.3), *oaz1a* (XM_015977278.3), *tbp* (XM_054731864.1), and *th* (XM_015964874.2) were designed using NCBI Primer Blast. *th* – F, 5′ CTCCCCAGAACCTGACTGTG 3′; R, 5′ CGTTTTGTTTGCAAAGGCCG 3′. RG sequences are listed in Table 1.*actb* primer sequences are published in [12]. We evaluated *tbp* primer sets described in Kabilijo et al. [25] and Baumgart et al. [15], but found non-specific amplification to our template cDNAs. Primer specificity was confirmed for all primer pairs by gel electrophoresis and single, distinct bands for each primer pair were present corresponding with the expected product size. Melt curve analysis was performed following all RT-qPCR reactions to additionally verify the absence of non-specific product amplification.

**Table 1:** RT-qPCR candidate reference genes. *Matsui et al. [12].

Gene	Function	Accession Number	Forward primer (5′ – 3′)	Reverse primer (5′ – 3″)	Primer Efficiency	Product Size
***actb***	Cytoskeleton structure	XM_015960326.3	GGCATCCTGACCCTGAAGTA	CACACGGAGCTCGTTGTAGA	101.44 %	102*
***cyc1***	Cellular respiration	XM_015958440.3	GAAAGTTGCCCTGACAACGC	GTAACCACGGCGAACACTTG	93.45 %	172
***gapdh***	Glycolysis	XM_015950201.3	GGGTGTCAACCACGAGAAGT	GTGAACCGTGCTCATCAGGC	92.69 %	139
***gusb***	Lysosomal degregation	XM_015961804.3	TCCTGCGTGATTACCACAGT	CTTTGGGTTGTCTTTGCCGAG	104.10 %	156
***oaz1a***	Protein synthesis	XM_015977278.3	TAGCAGTAACCCGTGTCCAG	CAACAACTGAGCATCGGAGTAG	98.40 %	139
***tbp***	Transcription initiation	XM_054731864.1	CACAGGTACCAGACCAAATGTTG	GTCAGCGGAGCGTTCTG	108.08 %	172

### Data analysis

Primer efficiencies were determined using the formula: $E=100*(10^{\frac{-1}{\mathrm{Slope}}}-1)$ , with slope calculation resulting from a standard curve generated with four 10-fold dilutions of cDNA plotted against corresponding Cq values. Relative fold change was calculated as in described in Pfaffl et al. [26], using the mean Cq of young (10 week post-hatching) samples of both sexes as a calibrator sample pool to generate delta Cq values. For normalization to two reference genes, we used the formula described in Vandesompele et al. [27] to calculate gene expression. Graphs were generated using R programming language and figures were assembled with Affinity Designer.

### Statistics

Each sex and age group evaluated contained three biological replicates per group. For analyses of Cq values, there were three technical replicates per biological replicate, resulting in a total sample size of nine per group. For fold change analyses, three technical replicates from each biological replicate were averaged for the basis of fold change calculation, resulting in a sample pool of three per group. We evaluated homogeneity of variance using Levene’s test and used the Shapiro-Wilk test to evaluate normality. If both assumptions were met, we used a two-way ANOVA with age and sex as fixed factors, followed by Tukey’s multiple comparison test for post hoc comparisons. If assumptions of homoscedasticity or normality were not met, we used a generalized least squares (GLS) approach for our model. Post hoc testing under this framework was conducted using estimated marginal means (emmeans) with Tukey adjustment for multiple comparisons. Statistical significance was defined as P < 0.05, corresponding to a 95 % confidence interval.

## Results

### Variation in reference gene Cq values by sex and age

We examined the stability of six candidate RGs (Table 1) at two different ages, 10 and 25 weeks post-hatching, in both male and female brains. For *actb*, we evaluated primers published in Matsui et al. [12]; for other genes, we designed the primer sequences as reported in the Methods. To assess stability, we compared mean Cq values between age and sex groups to identify any age- or sex-dependent variation in transcript abundance. We used equal masses of total RNA for each sample and all RG targets were amplified from the same cDNA sample set.

**Fig. 1:**
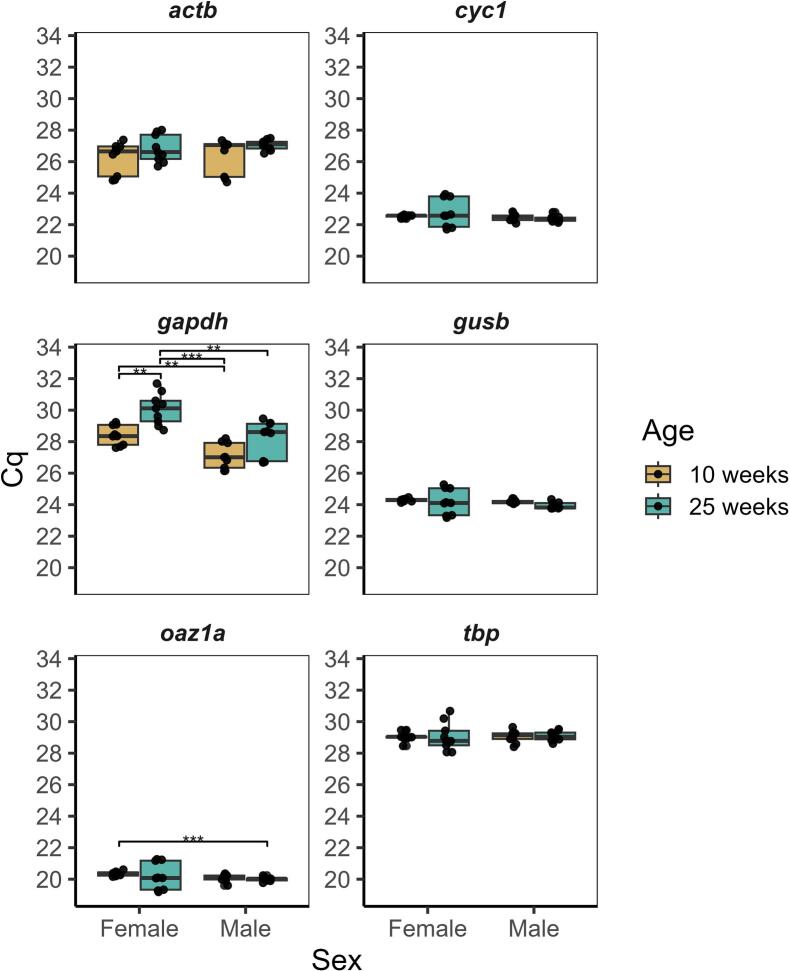
**Candidate reference gene Cq values.** Boxplots show the median and interquartile range (25th to 75th percentile) of unaltered Cq values. Whiskers extend to values 1.5 times the interquartile range. Individual data points are overlaid. Each datapoint represents one technical replicate (three per sample), with three samples per group. The same sample set was used for each reference gene analysis. Hypothesis tests were conducted as described in the methods section. Significance levels: P < 0.01 − **; P < 0.001 − ***.

As shown in Fig. 1, the mean Cq values of *actb, cyc1, gusb,* and *tbp* showed no significant age- or sex-dependent differences. In contrast, *gapdh* expression increased in aging females (P = 0.0045), but not in males, indicating a sex-specific difference in expression between young and old samples. *gapdh* expression also differed between the analogous groups in young (males vs. females) and again in older age groups, showing instability across age and sex. There was also a difference in overall *oaz1a* expression between young females and older females.

We also assessed the variability of expression within each group (i.e. how variable is *actb* expression in 10 weeks post-hatching female brains?). Variance around mean Cq values (Table 2) suggests that *cyc1*, *gusb*, and *oaz1a* expression is more consistent (lower variance) at 10 weeks post-hatching but increases in variability at 25 weeks post-hatching in females. *tbp* variance followed this same trend but was overall more variable (ranging from 0.0962 to 0.297 between young samples and older males and increasing to 0.812 in older female samples). *actb* and *gapdh* differed from this tendency, and generally had high variance across all groups, with *gapdh* showing the single highest variance (1.32 in 25-week males), an observation that is inconsistent with requirements for RG dynamics.

**Table 2:** Mean Cq Values and variance of mean for each reference gene.

	10-Week-Old Female		25-Week-Old Female		10-Week-Old Male		25-Week-Old Male	
Gene	Mean Cq	Variance	Mean Cq	Variance	Mean Cq	Variance	Mean Cq	Variance
***actb***	26.2	1.020	26.8	0.752	26.3	1.230	27.1	0.105
***cyc1***	22.5	0.086	22.7	0.805	22.5	0.053	22.4	0.037
***gapdh***	28.4	0.623	30.1	1.010	27.1	0.648	28.2	1.320
***gusb***	24.3	0.097	24.2	0.660	24.2	0.016	23.9	0.043
***oaz1a***	20.3	0.146	20.2	0.726	20.1	0.055	20.0	0.018
***tbp***	29.0	0.297	29.1	0.812	29.0	0.142	29.1	0.096

### Reference gene choice affects normalized TH expression

**Fig. 2:**
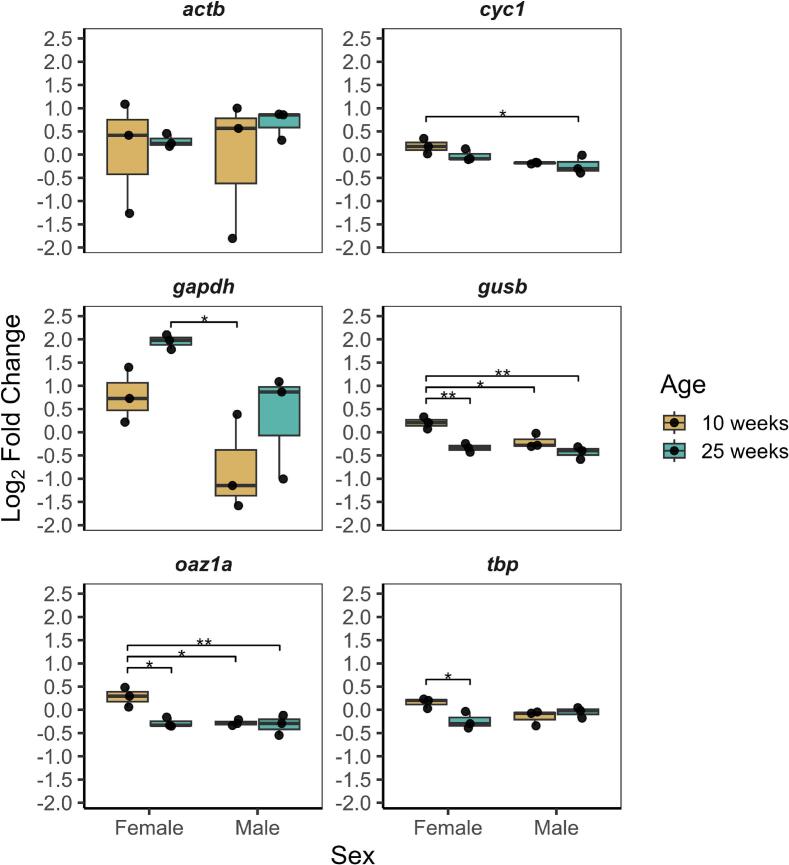
**Tyrosine hydroxylase (*th*) expression normalized to different reference genes.** The same *th* output was normalized to candidate reference genes. Boxplots show the median and interquartile range (25th to 75th percentile) of *th* fold change normalized to each reference gene. Reference genes used for normalization are indicated above each plot. Each point represents the mean of three technical replicates for one sample, with three samples per group. Significance levels: P < 0.05 − *; P < 0.01 − **;

As proof of concept, we evaluated the differences in calculated *th* fold change when expression is normalized to different reference genes with varying stability (Fig. 2). To assess the effects of age, sex, and their possible interaction, our analysis employed a two-way ANOVA.th expression differed between sexes when normalized to *cyc1* (F_1,8_ = 11.825, P = 0.0088), *gapdh* (F_1,8_ = 11.097, P = 0.0104), *gusb* (F_1,8_ = 11.076, P = 0.0104), and *oaz1a* (F_1,8_ = 10.057, P = 0.0132). Normalized *th* expression also changed across age, without regard to sex, when normalized to *gapdh* (F_1,8_ = 11.097, P = 0.0104), *gusb* (F_1,8_ = 27.967, P < 0.001), and *oaz1a* (F_1,8_ = 10.056, P = 0.0132). In Tukey post hoc comparisons between groups, we also found a decrease in *th* expression specific to female aging. When *th* expression was normalized to *gusb* (P = 0.0043), *oaz1a* (P = 0.0129), or *tbp* (P = 0.043), there was a decrease from young to old females, but no change across the male age groups tested. Additionally, when normalized to *gusb* and *oaz1a, th* expression was higher in young females compared to both male age groups.

In our analysis of *th* expression, we found significant age-sex interactions when *th* expression was normalized to *oaz1a* (F_1,8_ = 7.605, P = 0.025) and *tbp* (F_1,8_ = 8.787, P = 0.018). This suggests that *th* expression is interdependent on both age and sex when normalized to these two genes. Although not statistically significant, *th* expression normalized to *Gusb* age-sex interactions may require further exploration (F_1,8_ = 4.195, P = 0.075).

In contrast, *th* expression normalized to *actb, cyc1,* and *gapdh* did not have any significant age-sex interactions and generally differed from the trends observed in our other tested RGs. *actb*-normalized expression did not change across age or sex. *cyc1*-normalized expression only showed a significant difference between 10-week females and 25-week males (P = 0.0111), while *gapdh*-normalized expression showed a significant difference between 25-week females and 10-week post-hatching males (P = 0.0161). Taken together, these results demonstrate that reported outcomes of gene expression can vary drastically depending on reference gene selection. Additionally, these data reinforce the importance of validating RG stability in different experimental conditions.

### Computational assessment of reference gene stability

**Fig. 3:**
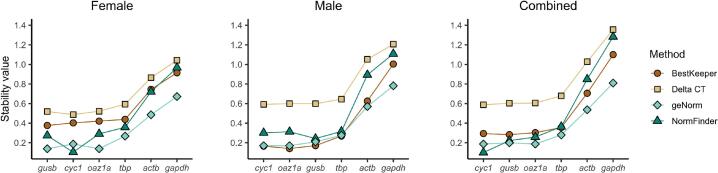
BestKeeper, Delta Ct, geNorm, and NormFinder evaluative methods summarized by RefFinder. From left to right, plots represent the same dataset analyzed separately for male and female samples, and then combined together. On each respective x-axis reference genes are ordered, from left to right, most stable to least stable. The y-axis shows the stability value defined by each method. A lower stability value indicates a more stable reference gene. These values were generated by RefFinder [28]. BestKeeper: standard deviation of Cq values [26]. Delta Ct: mean standard deviation of delta Ct between gene pairs across all samples [29]. GeNorm: average pairwise variation between a gene and all other candidate reference genes [27]. In geNorm, the two most stable genes are expected to have the same lowest value and are therefore ranked equally (two genes with the lowest stability value). NormFinder: estimate of intra- and inter-group variation in expression [30]. The results of this figure were used to generate the ranking shown in Table 3.

Several computational tools have been developed to assess RG stability under various experimental conditions. RefFinder [28] is a widely used program that integrates results from multiple RG-testing algorithms, including BestKeeper [26], Delta Ct [29], geNorm [27], and NormFinder [30] to produce a comprehensive best-to-worst stability ranking. For our analysis, we divided our RG expression dataset into three groups: Female, Male, and Combined. We sought to identify the best RG(s) for each sex in aging and, additionally, determine if stable genes in sex-specific sets would perform well in a mixed-sex context. Fig. 3 summarizes the results of each algorithm contained within RefFinder. Within each dataset (Male, Female, and Combined), RGs are ranked from most stable (left) to least stable (right). Lower stability values indicate higher stability attributed to the RG by each computational method. RefFinder assigns a numeric weight to these methods and averages them to get a definitive ranking, shown in Table 3. The top three RGs across all datasets were *cyc1*, *gusb*, and *oaz1a*, with the rank order dependent on sex. The least stable RG in aging, regardless of sex, was *gapdh*, followed by *actb*.

**Table 3:** RefFinder top three reference genes for the female, male, and combined dataset.

Female	Male	Combined
1. *gusb*	1. *cyc1*	1. *cyc1*
2. *cyc1*	2. *oaz1a*	2. *gusb*
3. *oaz1a*	3. *gusb*	3. *oaz1a*

### TH expression normalization to top reference genes pairs

**Fig. 4:**
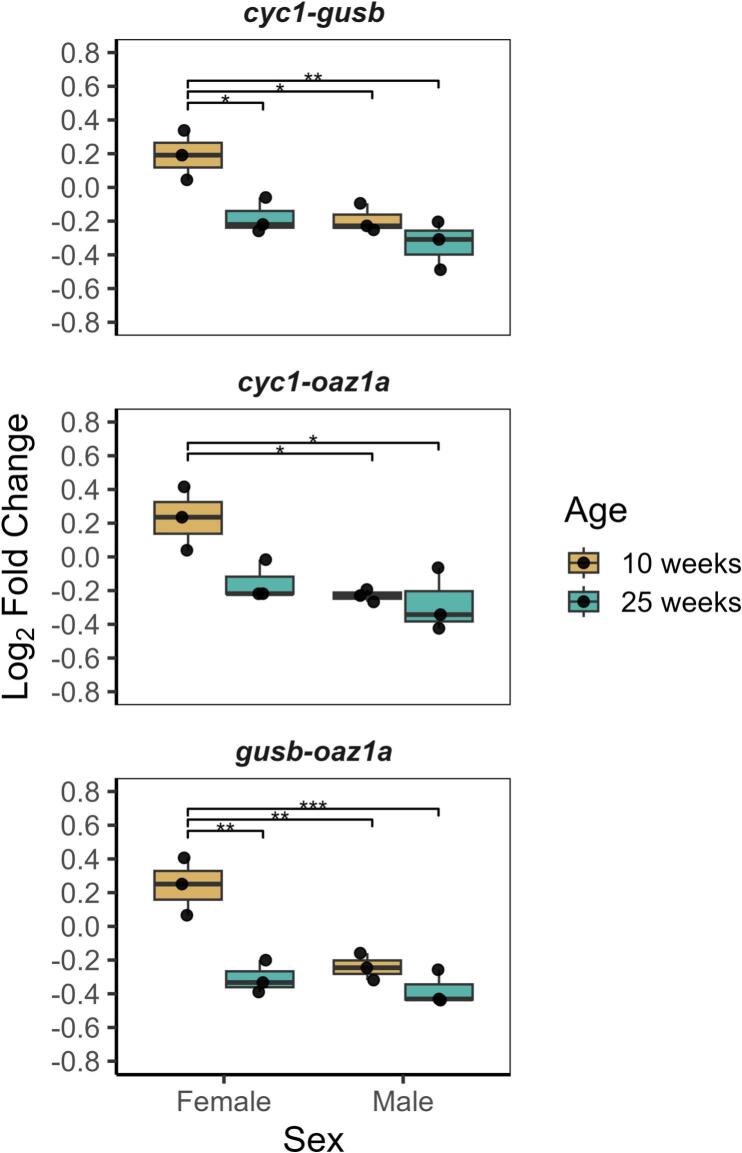
***TH* expression normalized to top two combinations of reference genes.** We normalized the same *th* expression set to the top two RefFinder-identifed genes for males (*cyc1*-*oaz1a*), females (*cyc1*-*gusb*), and the top two genes for females identified by geNorm (*gusb*-*oaz1a*), also ranked highly in all datasets**.** Boxplots show the median and interquartile range (25th to 75th percentile) of *th* fold change normalized to each reference gene combination. Significance levels: P < 0.05 − *; P < 0.01 − **; P < 0.001 − ***.

In the normalization strategy established by Vandesompele et al. [27], target gene expression is normalized to the geometric mean of multiple reference genes rather than a single gene, as in the comparative delta Ct method [29]. This approach improves reliability by reducing the influence of outliers and minimizing the risk that variability in a single reference gene will skew results. We re-examined *th* fold change between young and old samples (Fig. 4) but now normalizing to the top reference genes for each sex: 1) *cyc1* and *gusb* (top ranked in the female and combined dataset), 2) *cyc1* and *oaz1a* (top ranked in the male dataset), and 3) *gusb* and *oaz1a* (ranked highly across all groups). Overall trends were similar between the three groups, with slight differences depending on the RG pair used. Outside of changes in significance levels, the main difference between the three RG pairs used was that *th* expression normalized to *cyc1-oaz1a* did not show a significant decrease between young and old female brains (P = 0.0511). Importantly, *cyc1* and *oaz1a* are the top two identified genes for the male dataset, not the female dataset, highlighting differences between gene expression measurements with alternate RG pairs. In the other pairs tested, each ranked as the top two genes for females by RefFinder or GeNorm, *th* expression decreased significantly in females from young-to-old brains. In aging male brains, there was no change in *th* expression, regardless of the normalization RG combination used. These findings indicate that our RefFinder-based analysis led to similar results in both males and females and reinforces our reference gene selection.

## Discussion

In our evaluation of six candidate reference genes – *actb*, *cyc1*, *gapdh*, *gusb*, *oaz1a*, and *tbp* – we identified *cyc1*, *gusb*, and *oaz1a* as the most stable reference genes for RT-qPCR normalization in the aging *N. furzeri* brain across both sexes. Although *actb* and *gapdh* are widely used for normalization in gene expression studies, we found high variation, with *gapdh* in particular showing age- and sex-dependent changes. Additionally, we found that reference gene normalization to *th*, a gene expected to decrease with age in the *N. furzeri* brain, revealed significant age-sex interactions highly dependent on the reference gene used. Interestingly, the decline in *th* expression was observed only within the female age groups (Fig. 2 and Fig. 4). One possible explanation is that the apparent age-related decline in females reflects a difference in the timing of *th* expression between sexes. Males may undergo an earlier decline in *th* (prior to 10 weeks), while females exhibit a later decline (closer to 25 weeks). Although this is speculative, it is consistent with preliminary findings (manuscript in preparation) and with evidence from Parkinson’s disease analyses where females exhibit increased neuroprotection compared to males [31,32]. Regardless, while our primary goal was to identify stable reference genes, our findings highlight a potential sex-specific component of brain aging in *N. furzeri* that warrants further study.

Several *N. furzeri* studies have used RT-qPCR, with normalization to reference genes that were evaluated in our study. For example, *th* expression normalized to *actb* resulted in a significant decrease in expression from 1 to 5 months of age [12]. While we did not find overall age- or sex-dependent changes in *actb* or in *actb*-normalized expression, we did find high variability in *actb* Cq values. Additionally, RefFinder ranked it among the least stable RGs that we tested, following *gapdh*. However, in this case, selection of the RG likely does not affect conclusions, particularly because the authors used multiple techniques to verify their findings. Additionally, only male fish were used for analyses, and there could be sex-specific differences. In another example of *N. furzeri* RT-qPCR-based studies, expression of *hdac1* in the brain was normalized to *tbp* [33]. *tbp* used as reference gene in our study resulted in significant age-sex interactions for *th*-normalized expression. With this in mind, it raises the question of possible sex-dependent differences in aging *hdac1* expression that were not detectable, as only male fish were included in the study. In the context of serotonin signaling, *polr2eb* was found to be a suitable reference gene across age and sex because it does not have any age, sex, or interaction effects [34]. This supports the importance of reference gene validation, and that age and sex should be considered as factors in gene expression studies. Other *N. furzeri* analyses not focused on age-related expression used *actb* and *gapdh* as reference genes as in brain RT-qPCR normalization [35]. In that and similar contexts, RG age-sex instability may not be a concern, but these variables should be evaluated directly to rule out any confounding influences on experimental conclusions.

Although we identified several stable candidate reference genes, we emphasize that validation of reference gene(s) within the specific context of an experiment is essential. As seen in our results, factors such as age and sex can influence both reference gene expression and target gene normalization. Other experimental factors, such as drug treatments, could result in changes to gene expression, including reference genes, and additional controlled variables such as housing conditions, fish strain, and genetic background should all be considered to prevent variability across studies. In our study, we acknowledge the limitation of a relatively small sample size, and increasing sample size could impact effect sizes and corresponding statistical outcomes. Nonetheless, we believe that identifying these candidate reference genes provides a foundation for improving gene expression analyses and increases reliability in future studies. Additionally, our work reinforces the need to base conclusions on multiple reference genes and sets a foundation for investigating sex-specific neurodegeneration in *N. furzeri*.

## Author Contributions

All authors contributed to the study conception and design. Material preparation, data collection and analysis were performed by Emily Whisenant. The first draft of the manuscript was written by Emily Whisenant and all authors commented on previous versions of the manuscript. All authors read and approved the final manuscript.

## Significance Statement

Reliable reference genes are essential for measuring gene expression changes via RT-qPCR, yet their stability can vary with both age and sex. In the African turquoise killifish, we show that reference gene choice strongly influences the ability to detect age-related changes in the brain. Additionally, we observed sex-specific differences in aging, highlighting the need to account for both biological sex and reference gene stability in studies of aging.

## Funding declaration

The authors have no funding sources to declare.

## CRediT authorship contribution statement

**Emily Whisenant:** Writing – original draft, Methodology, Investigation, Formal analysis, Data curation, Conceptualization. **Arne C. Lekven:** Writing – review & editing, Supervision, Resources, Project administration, Formal analysis, Conceptualization.

## Declaration of competing interest

The authors declare the following financial interests/personal relationships which may be considered as potential competing interests: The authors declare no financial or non-financial interests related to this work. *N. furzeri* experiments in the Lekven lab are approved by the University of Houston IACUC.
